# Application of Clinical Department–Specific AI-Assisted Coding Using Taiwan Diagnosis-Related Groups: Retrospective Validation Study

**DOI:** 10.2196/59961

**Published:** 2025-02-12

**Authors:** An-Tai Lu, Chong-Sin Liou, Chia-Hsin Lai, Bo-Tsz Shian, Ming-Ta Li, Chih-Yen Sun, Hao-Yun Kao, Hong-Jie Dai, Ming-Ju Tsai

**Affiliations:** 1 Department of Healthcare Administration and Medical Informatics, Kaohsiung Medical University, No.100, Shih-Chuan 1st Road, Sanmin Dist, Kaohsiung, 807, Taiwan, 886 73121101 ext 2648; 2 Department of Medical Records, Kaohsiung Medical University Hospital, Kaohsiung, Taiwan; 3 Intelligent System Lab, College of Electrical Engineering and Computer Science, Department of Electrical Engineering, National Kaohsiung University of Science and Technology, Kaohsiung, Taiwan; 4 Department of Information Technology, Kaohsiung Medical University Hospital, Kaohsiung, Taiwan; 5 Department of Electrical Engineering, National Kaohsiung University of Science and Technology, Kaohsiung, Taiwan; 6 School of Post-Baccalaureate Medicine, Kaohsiung Medical University, Kaohsiung, Taiwan; 7 National Institute of Cancer Research, National Health Research Institutes, Tainan, Taiwan; 8 Center for Big Data Research, Kaohsiung Medical University, Kaohsiung, Taiwan; 9 Division of Pulmonary and Critical Care Medicine, Department of Internal Medicine, Kaohsiung Medical University Hospital, Kaohsiung, Taiwan; 10 Department of Internal Medicine, School of Medicine, College of Medicine, Kaohsiung Medical University, Kaohsiung, Taiwan

**Keywords:** diagnosis-related group, artificial intelligence coding, *International Classification of Diseases, Tenth Revision, Clinical Modification*, *ICD-10-CM*, coding professionals

## Abstract

**Background:**

The accuracy of the *ICD-10-CM* (*International Classification of Diseases, Tenth Revision, Clinical Modification*) procedure coding system (PCS) is crucial for generating correct Taiwan diagnosis-related groups (DRGs), as coding errors can lead to financial losses for hospitals.

**Objective:**

The study aimed to determine the consistency between an artificial intelligence (AI)-assisted coding module and manual coding, as well as to identify clinical specialties suitable for implementing the developed AI-assisted coding module.

**Methods:**

This study examined the AI-assisted coding module from the perspective of health care professionals. The research period started in February 2023. The study excluded cases outside of Taiwan DRGs, those with incomplete medical records, and cases with Taiwan DRG disposals *ICD-10* (*International Statistical Classification of Diseases, Tenth Revision)* PCS. Data collection was conducted through retrospective medical record review. The AI-assisted module was constructed using a hierarchical attention network. The verification of the Taiwan DRGs results from the AI-assisted coding model focused on the major diagnostic categories (MDCs). Statistical computations were conducted using SPSS version 19. Research variables consisted of categorical variables represented by MDC, and continuous variables were represented by the relative weight of Taiwan DRGs.

**Results:**

A total of 2632 discharge records meeting the research criteria were collected from February to April 2023. In terms of inferential statistics, κ statistics were used for MDC analysis. The infectious and parasitic diseases MDC, as well as the respiratory diseases MDC had κ values exceeding 0.8. Clinical inpatient specialties were statistically analyzed using the Wilcoxon signed rank test. There was not a difference in coding results between the 23 clinical departments, such as the Division of Cardiology, the Division of Nephrology, and the Department of Urology.

**Conclusions:**

For human coders, with the assistance of the *ICD-10-CM* AI-assisted coding system, work time is reduced. Additionally, strengthening knowledge in clinical documentation enables human coders to maximize their role. This positions them to become clinical documentation experts, preparing them for further career development. Future research will apply the same method to validate the *ICD-10* AI-assisted coding module.

## Introduction

The *International Statistical Classification of Diseases* (*ICD*) system was set up by the World Health Organization (WHO) for the purpose of tracking diseases globally. Over the past several decades, the WHO has made significant changes to both content and structure. It accompanies a new scientific understanding of diseases and new structures for organizing *ICD* codes to accommodate enhanced use and extensibility [1]. The WHO introduced the *ICD* in 1948, and it is a universal language used to categorize diseases or causes of death. The use of it is attributed to health care–related units in 194 countries and is generated by professional coders based on discharge records, with countries adjusting the *ICD* to their circumstances. In 2016, Taiwan adopted the international trend of switching from *ICD-9-CM* (*International Classification of Diseases, Ninth Revision, Clinical Modification*) to *ICD-10-CM* (*International Classification of Diseases, Tenth Revision, Clinical Modification*) procedure coding system (PCS) for coding hospital patient diagnoses, procedures, analysis and reimbursement. The National Health Insurance Administration (NHIA) under the Ministry of Health and Welfare has adopted the 2014 edition of *ICD-10-CM* PCS, with approximately 71,900 diagnosis codes and 78,500 procedure codes.

The use of the *ICD-10-CM* PCS involves coding and classifying morbidity data from health records, reimbursement claims, and administrative databases. Improving health care quality, monitoring public health, and conducting research are all benefits of the *ICD-10-CM* PCS in Taiwan and involves converting the physician’s discharge diagnosis into *ICD-10-CM* codes by following the primary diagnosis selection principle announced by the NHIA. The diagnosis-related group (DRG) provides information such as health insurance reimbursement, relative weight, presence of comorbidities, and complications for the current hospitalization.

The accuracy of *ICD-10-CM* PCS coding is crucial for generating accurate Taiwan DRGs, as coding errors can lead to financial losses for hospitals [2,3]. According to the coding principles set forth by the NHIA and the Taiwan Society of Medical Records Management, coding is based on the inpatient and emergency room records of patients. In the past, this task was undertaken by trained and certified clinical coding professionals (referred to as coding professionals hereafter), but with the rapid advances of medical technology, the rules of disease classification have also evolved, and coding professionals must regularly accumulate relevant training hours to update their disease classification skills [4].

In recent years, artificial intelligence (AI) and natural language processing have shown exciting potential in the field of automatic clinical coding. In 2021, the disease coding scales in the United States were worth approximately 18 billion US dollars. Several technology companies in the United States have developed AI-assisted coding systems, and scholars believe that interdisciplinary collaboration and feedback from clinical coding professionals are essential to further refine the modules [5,6]. Research on AI-assisted coding consistently conclude that it improves quality and reduces error rates while saving costs [7,8]. AI-assisted *ICD-10-CM* PCS coding can be considered as a text classification task within the field of machine learning [9]. In recent years, studies in the machine learning text classification field have predominantly proposed using deep learning–based neural networks [10]. Many research papers have focused on AI assistance in *ICD-10* (*International Statistical Classification of Diseases, Tenth Revision)* coding [11-14], but few have examined the results of coding implementation from the perspective of disease classification personnel. The development and validation process of the AI-assisted coding model requires the involvement and feedback of clinical coders to enhance accuracy and correctness, aligning with user needs [15].

In Taiwan, several hospitals have also ventured into the development of AI-assisted coding for disease classification. However, due to variations in physicians’ documentation of medical records across different hospitals, the AI-assisted coding systems developed are not universally applicable [11], necessitating the development and validation of customized AI-assisted coding systems. Medical coding personnel must review the discharge records meticulously and then translate the discharge diagnoses and procedures (interventions) recorded in the medical records into *ICD-10* codes. In the past, the most significant factor contributing to coding errors was handwritten medical records by physicians, which were difficult to decipher or included abbreviations, leading to mistakes [16]. In recent years, most medical centers in Taiwan have adopted electronic health records, resulting in a significant reduction in coding errors caused by handwritten records. Clinical coding personnel also encounter various pressures, including the need to accomplish all inpatient coding tasks within specified deadlines, optimize Taiwan DRGs assignment coding, enhance and maintain coding reliability and validity, and engage in discussions with clinical physicians regarding the content of medical record writing.

Recently, the global trend in AI coding has been on the rise [11,13,17-19]. In this study, we have developed an exclusive *ICD-10-CM* AI-assisted coding module. Coding professionals took part in the research and offered suggestions to improve the efficiency of coding operations. Consequently, this study focuses primarily on the following two research aims: (1) to verify the consistency between the AI-assistant coding module and a coding professional in encoding, based on the MDC results in Taiwan DRGs and (2) to find the clinical departments within the medical center that can benefit from using the developed AI-assisted coding module.

## Methods

### Data Description

This study used a total of 136,841 unstructured discharge summaries of patients who were hospitalized, recorded in Kaohsiung Medical University Chung-Ho Memorial Hospital from April 1, 2019, to December 31, 2020, as the primary data source. Textbox 1 displays an example of a discharge summary from the Kaohsiung Medical University Chung-Ho Memorial Hospital.

Textbox 1. **Example discharge summary.**

**Chief complaint:**

Abdominal pain for 1 day

**Impression on admission:**

# Sepsis, focus on retroperitoneal abscess

**Discharge diagnosis:**

# Sepsis, focus on retroperitoneal abscess due to surgical site infection

**History on admission:**

This time, according to the patient's statement, he suffered from recurrent abdominal dull pain after discharge. The pain was serious by jejunostomy feeding, and there was no relieving factor. The pain suddenly progressed...

This study verified the AI-assisted coding from the perspective of coding professionals. Since the AI-assisted coding system was introduced in the medical center in February 2023, the study period began in February 2023. The subjects of this study were selected based on the following exclusion criteria: non–Taiwan DRGs cases, cases with procedures (*ICD-10* PCS) in Taiwan DRGs, and cases with incomplete medical records. According to the study conditions, there were approximately 700 to 1000 cases per month. The coding by both the AI-assisted coding module and coding professionals were based on the electronic discharge summaries of a certain medical center each month.

### Research Design

After each data entry was encoded by the AI-assisted coding module and verified by a coding professional, it was transmitted to a certain university’s database. The results of both the AI-assisted coding and coding professional were compared using an Excel (Microsoft) file. Following the linkage to the NHIA’s DRG calculation software, separate datasets for Taiwan DRGs were obtained for both the AI-assisted coding and coding personnel, with the consistency of the primary diagnosis coding between these two groups being examined. In cases of discrepancies, the medical records were scrutinized again by the coding professional to determine if the AI-assisted coding results met the criteria for primary coding as per the coding professional; the consistency results of the Taiwan DRGs data for both the AI-assisted coding and coding professional were adjusted accordingly.

### AI-Assisted Coding Construction Process

The AI-assisted *ICD-10-CM* coding system was developed by CSL, CHL, and BTS, based on approximately 110,000 discharge summaries collected from April 1, 2019 to December 31, 2020, in a medical center. The deidentified summary data were processed by segmenting sentences and filtering out meaningless delimiters and prefix symbols (eg, # or "") by using a clinical natural language processing tool [20]. The data were categorized into 21 groups based on the first 3 codes of the *ICD-10-CM*, and models were built using bidirectional encoder representations from transformers (BERTs) [21] and hierarchical attention networks (HANs) [22]. The results favored HANs, leading to the decision to adopt the HAN module. The precision, recall, and F1 scores of the developed HAN model were 0.55, 0.82, and 0.66, respectively. For the top 50 most frequent codes, the F1 score of the developed HAN model was 0.818.

**Figure 1.**
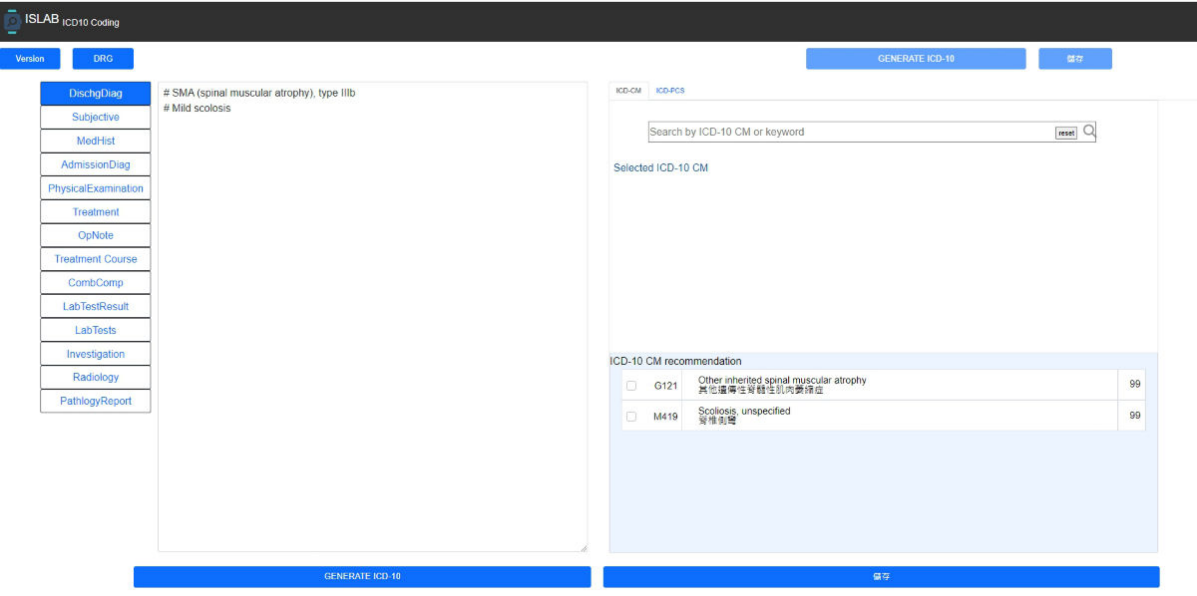
User interface screenshot.

Aside from module modeling, another time-consuming task was the design of the user interface for the coding professionals, as it needed to present discharge summaries, laboratory data, and imaging reports, as well as the *ICD-10-CM* codes predicted by the AI-assisted coding module. Coding professionals were actively involved in providing feedback during the interface design process. Figure 1 provides an illustration of the designed user interface, which provided suggestions automatic *ICD-10-CM* recommendation and fields for coding professionals to input the final codes. The developed AI-coding system was integrated into a medical center’s hospital information systems in November 2022 and operated in February 2023.

### Ethical Considerations

This study was approved by Kaohsiung Medical University Ching-Ho Memorial Hospital (institutional review boards number: KMUHIRB-E(II)-20230214). The institutional review board approval covered secondary analysis without additional consent. Data was anonymized or deidentified. There was not any compensation provided to participants.

### Statistical Analysis

The study involved an analysis incorporating descriptive statistics for exploration, as well as inferential statistics for investigating MDCs and relative weight. Statistical computations were conducted using SPSS version 19. Research variables consisted of categorical variables represented by MDC, and continuous variables were represented by the relative weight of Taiwan DRGs.

## Results

### Distribution of *ICD-10* Codes

The distribution of the *ICD-10* codes seen in the collected training dataset is shown in Multimedia Appendix 1. The first digit of the *ICD-10-CM* code consisted of English letters, so the alphabetical characters on the horizontal axis of the log data were the first digit of the *ICD-10-CM* code, showing diseases pertaining to different systems. According to Multimedia Appendix 1, data starting with codes C, E, and I in *ICD-10-CM* had the highest volume, with C representing neoplastic diseases; E for endocrinal, nutritional, and metabolic diseases; and I for diseases of the circulatory system. These were the body systems with the highest learning volumes for the AI-assisted coding module.

### Descriptive Statistics

In the period from February to April 2023, a total of 15,756 discharges were recorded. Excluding cases with interventions, non–Taiwan DRG cases, and cases with incomplete medical records, there was a total of 2632 cases. The primary diagnosis was the key factor in deciding the main disease category, while secondary diagnoses only affected the distribution of Taiwan DRGs within the same primary disease category. According to disease classification rules, the primary diagnosis was based on the reason for the patient’s admission, but only one disease could be selected as the primary diagnosis. If multiple diseases were treated during admission, selecting any one of them as the primary diagnosis was not considered an error. Therefore, the coding professional (author ATL) manually examined the discharged cases’ notes to categorize the output of the AI-assisted system into one of the following categories. The results are shown in Table 1.

**Table 1.** Frequency distribution and percentage analysis of primary diagnoses.

Variable	Month of case, n (%)		
	February (n=748)	March (n=991)	April (n=893)
No primary diagnosis	181 (24.2)	277 (28)	285 (31.9)
Incorrect secondary diagnosis with a primary diagnosis	462 (61.8)	477 (48.1)	369 (41.3)
All correct	79 (10.6)	181 (18.3)	177 (19.8)
All incorrect	26 (3.5)	56 (5.7)	62 (7)

Operational definitions were as follows:

- No primary diagnosis: in comparison to the coding professional, a single hospitalization’s predicted diagnosis codes did not include a primary diagnosis.
- Incorrect secondary diagnosis with a primary diagnosis: in comparison to the coding professional, a single hospitalization’s predicted diagnosis codes included a primary diagnosis, but there was at least 1 error in the secondary diagnoses.
- All correct: all predicted diagnosis codes for a single hospitalization perfectly aligned with those given by the coding professional.
- All incorrect: in comparison to the disease classification personnel, none of the predicted diagnosis codes in a single hospitalization were the same.

**Figure 2.**
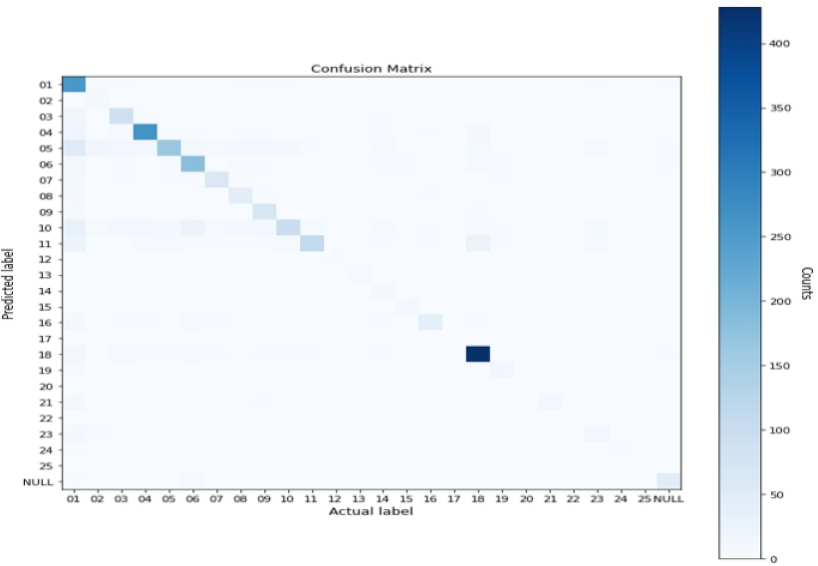
MDC heat map analysis between AI-coding module and professionals.

In Figure 2, we analyzed the agreement of MDC classification between the AI-assisted coding module and the coding professional through a heat map analysis. The vertical and horizontal axes in Figure 2 represent MDCs coded by the AI-assisted coding module and MDCs coded by coding professionals, respectively. The intensity of color in the figure indicated a higher number of agreed MDCs between the AI-assisted coding module and professionals. As shown in Figure 2, MDC 1 (diseases and disorders of the nervous system), MDC 4 (diseases and disorders of the respiratory system), and MDC 18 (infectious and parasitic diseases and disorders) had the highest agreements.

### The κ Coefficient Test

Furthermore, we assessed the MDC agreement between the AI coding module and coding professionals using the κ coefficient test. The κ values were broadly categorized into 5 groups based on various levels of agreement: extremely low agreement (0.00-0.20), fair agreement (0.21-0.40), moderate agreement (0.41-0.60), high agreement (0.61-0.80), and almost perfect agreement (0.81-1.0).

When analyzing the cumulative data for February to April 2023 (Table 2), the MDCs with the highest consistency were MDC 4 (diseases and disorders of the respiratory system) and MDC 18 (infectious and parasitic diseases and disorders), followed by MDC 1 (diseases and disorders of the nervous system), MDC 3 (diseases and disorders of the ear, nose, mouth and throat), MDC 6 (diseases and disorders of the digestive system), MDC 7 (diseases and disorders of the hepatobiliary system and pancreas), MDC 9 (diseases and disorders of the skin, subcutaneous tissue and breast), MDC 11 (diseases and disorders of the kidney and urinary tract), MDC 13 (diseases and disorders of the female reproductive system), MDC 15 (newborn and other neonates), and MDC 16 (diseases and disorders of the blood and blood forming organs and immunological disorders).

**Table 2.** Kappa tests for aggregation of major diagnostic category in the total counts for February to April 2023 (κ=0.592).

No.	Major diagnostic category	AI-assisted case coding (n=2362), n (%)	Cases coded by human coders (n=2362), n (%)	Kappa value
1	Diseases and disorders of the nervous system	280 (10.6)	509 (19.3)	0.670^a^
2	Diseases and disorders of the eye	9 (0.3)	38 (1.4)	0.300
3	Diseases and disorders of the ear, nose, mouth, and throat	113 (4.3)	132 (5)	0.689^a^
4	Diseases and disorders of the respiratory system	309 (11.7)	302 (11.5)	0.845^b^
5	Diseases and disorders of the circulatory system	310 (11.8)	184 (7)	0.607
6	Diseases and disorders of the digestive system	217 (8.2)	229 (8.7)	0.775^a^
7	Diseases and disorders of the hepatobiliary system and pancreas	90 (3.4)	84 (3.2)	0.710^a^
8	Diseases and disorders of the musculoskeletal system and connective tissue	66 (2.5)	87 (3.3)	0.576
9	Diseases and disorders of the skin, subcutaneous tissue, and breast	83 (3.2)	116 (4.4)	0.692^a^
10	Diseases and disorders of the endocrine, nutritional, and metabolic systems	237 (9)	132 (5)	0.505
11	Diseases and disorders of the kidney and urinary tract	205 (7.8)	120 (4.6)	0.648^a^
12	Diseases and disorders of the male reproductive system	7 (0.3)	3 (0.1)	0.362
13	Diseases and disorders of the female reproductive system	8 (0.3)	8 (0.3)	0.749^a^
14	Pregnancy, childbirth and puerperium	11 (0.4)	41 (1.6)	0.419
15	Newborn and other neonates (perinatal period)	9 (0.3)	15 (0.6)	0.635^a^
16	Diseases and disorders of the blood and blood forming organs and immunological disorders	64 (2.4)	57 (2.2)	0.624^a^
17	Myeloproliferative diseases and disorders (poorly differentiated neoplasms)	3 (0.1)	2 (0.1)	0.399
18	Infectious and parasitic diseases and disorders	465 (17.7)	505 (19.2)	0.870^b^
19	Mental diseases and disorders	23 (0.9)	0 (0)	—^c^
20	Alcohol or drug abuse or induced mental disorder	2 (0.1)	0 (0)	—
21	Injuries, poison, and toxic effects of drugs	24 (0.9)	21 (0.8)	0.404
22	Burns	0 (0)	1 (0)	—
23	Factors influencing health status and other contacts with health services	24 (0.9)	42 (1.6)	0.366
24	Multiple significant trauma	6 (0.2)	4 (0.2)	0.599
25	HIV infection	2 (0.1)	0 (0)	—
—	None	65 (2.5)	0 (0)	—

a High agreement (0.61-0.80).

b Almost perfect agreement (0.81-1.00).

c Kappa value was not calculated when there were 0 cases in a coding group.

### Inferential Statistical Analysis: Wilcoxon Signed Rank Test

The κ coefficient test was used for a broad-scale analysis of MDCs. However, under the same MDC, it was possible to further classify the data into numerous Taiwan DRGs, with each having its own code and relative weight. Even within the same MDC, this might result in different Taiwan DRGs. Furthermore, some diseases could be treated across departments. Therefore, for the statistical analysis of relative weight, we first conducted a normality analysis of the relative weights obtained from both AI-assisted coding and coding professionals. The statistical results based on the Kolmogorov-Smirnov analysis yielded a significance level of less than .05, showing a nonnormal distribution. Given that the research sample consisted of paired data, the nonparametric Wilcoxon signed rank test was used to analyze whether there were differences in relative weight between AI-assisted coding and coder-assigned coding; the null hypothesis assumed that there was no difference in relative weight between AI-assisted coding and coder-assigned coding.

The Wilcoxon signed rank test, with clinical departments as the unit of analysis, identified differences in relative weight in the following 12 departments: Division of Endocrinology and Metabolism, Division of Hematology and Oncology, Division of General Internal Medicine, Division of Geriatrics and Gerontology, Division of Trauma, Division of Neurosurgery, Division of Cardiovascular Surgery, Division of General and Digestive Surgery, Division of Pediatric Neurology, Department of Otorhinolaryngology, Department of Neurology, and Department of Rehabilitation Medicine. As shown in Table 3, the overall statistical result with a *P* value of <.001 showed that there were still differences between AI-assisted coding and coder-assigned coding in this study.

**Table 3.** Wilcoxon signed rank test results across various clinical departments (*P*<.001).

Clinical department	Frequency of cases (n=2632), n (%)	Relative weight (95%CI)		*P* value
		AI^a^ coding	Human coding	
Division of Gastroenterology	61 (2.3)	0.66 (0.58‐0.74)	0.70 (0.63‐0.77)	.12
Division of Hepatobiliary and Pancreatic Medicine	81 (3.1)	0.69 (0.63‐0.75)	0.74 (0.69‐0.79)	.07
Division of Cardiology	177 (6.7)	0.69 (0.65‐0.73)	0.72 (0.68‐0.75)	.39
Division of Chest Medicine	181 (6.9)	0.90 (0.85‐0.93)	0.90 (0.87‐0.94)	.50
Division of Nephrology	64 (2.4)	0.71 (0.65‐0.78)	0.71 (0.64‐0.78)	.91
Division of Endocrinology and Metabolism	36 (1.4)	0.62 (0.54‐0.71)	0.68 (0.59‐0.77)	.03
Division of Hematology and Oncology	42 (1.6)	0.79 (0.69‐0.88)	0.87 (0.80‐0.94)	.006
Division of Rheumatology, Immunology, and Allergology	36 (1.4)	0.68 (0.58‐0.79)	0.71 (0.64‐0.79)	.33
Division of Infectious Diseases	78 (3)	0.91 (0.85‐0.98)	0.94 (0.90‐0.99)	.39
Division of General Internal Medicine	205 (7.8)	0.69 (0.65‐0.73)	0.73 (0.69‐0.77)	.005
Division of Geriatrics and Gerontology	54 (2.1)	0.92 (0.85‐0.99)	0.99 (0.94‐1.04)	.003
Division of Trauma	16 (0.6)	0.43 (0.21‐0.65)	0.64 (0.46‐0.83)	.04
Division of Neurosurgery	151 (5.7)	0.53 (0.48‐0.58)	0.68 (0.63‐0.73)	<.001
Division of Cardiovascular Surgery	24 (0.9)	0.67 (0.54‐0.80)	0.94 (0.82‐1.06)	.002
Division of Chest Surgery	14 (0.5)	0.56 (0.43‐0.69)	0.60 (0.45‐0.75)	.40
Division of Pediatric Surgery	9 (0.3)	0.45 (0.31‐0.59)	0.48 (0.33‐0.63)	.28
Division of Plastic Surgery	9 (0.3)	0.64 (0.38‐0.90)	0.68 (0.44‐0.92)	.89
Division of Colorectal Surgery	46 (1.7)	0.54 (0.45‐0.62)	0.59 (0.52‐0.66)	.16
Division of Breast Oncology and Surgery	16 (0.6)	0.47 (0.32‐0.62)	0.62 (0.48‐0.75)	.05
Division of General and Digestive Surgery	56 (2.1)	0.56 (0.49‐0.62)	0.63 (0.57‐0.68)	.009
Department of Gynecology Obstetrics	60 (2.3)	0.47 (0.39‐0.55)	0.46 (0.40‐0.52)	.72
Division of Pediatric Hematology and Oncology	42 (1.6)	0.45 (0.38‐0.52)	0.47 (0.40‐0.55)	.44
Division of Pediatric Cardiology and Pulmonology	86 (3.3)	0.52 (0.37‐0.66)	0.53 (0.43‐0.63)	.24
Division of Pediatric Neurology	92 (3.5)	0.43 (0.34‐0.52)	0.45 (0.40‐0.50)	.008
Division of Neonatology	14 (0.5)	0.87 (0.28‐1.47)	0.81 (0.43‐1.18)	.69
Division of General Pediatrics	299 (11.4)	0.39 (0.36‐0.41)	0.39 (0.37‐0.41)	.56
Division of Pediatric Allergy Immunology	8 (0.3)	0.31 (0.17‐0.46)	0.35 (0.25‐0.46)	.32
Department of Otorhinolaryngology	53 (2)	0.57 (0.50‐0.63)	0.51 (0.45‐0.58)	.02
Ophthalmology Department	12 (0.5)	0.42 (0.34‐0.50)	0.44 (0.36‐0.52)	.44
Department of Orthopaedics	13 (0.5)	0.40 (0.25‐0.55)	0.52 (0.39‐0.65)	.16
Department of Urology	46 (1.7)	0.63 (0.56‐0.70)	0.62 (0.55‐0.69)	.95
Department of Dermatology	87 (3.3)	0.42 (0.37‐0.48)	0.41 (0.35‐0.46)	.18
Department of Neurology	366 (13.9)	0.66 (0.63‐0.69)	0.72 (0.69‐0.75)	<.001
Division of Family Medicine	49 (1.9)	0.96 (0.87‐1.04)	1.00 (0.93‐1.07)	.17
Department of Rehabilitation Medicine	47 (1.8)	0.85 (0.75‐0.94)	1.21 (1.14‐1.28)	<.001
Department of Psychiatry	1 (0)	—	—	—
Division of Oral Maxillofacial Surgery	1 (0)	—	—	—

a AI: artificial intelligence.

## Discussion

### Principal Results

For clinical coders, it is clear from the MDCs that AI-assisted coding can serve as a reference for disease systems. However, hospital administrators may require detailed statistical results from clinical departments to make judgments. In the individual clinical department analysis based on the Wilcoxon signed rank test, the Division of General Internal Medicine, the Department of Neurology, and the Division of Neurosurgery had the highest number of cases studied, but the statistical results were inconsistent with coder-assigned coding. However, in the κ coefficient test, the statistical results for the nervous system MDC were highly consistent. This is because patients admitted to the Department of Neurology and Neurosurgery do not exclusively have neurological conditions. According to further analysis shown in Table 4, the AI model’s predictions for neurological system diseases were still highly consistent with those of the disease classification staff in the neurology department. However, respiratory and urinary system conditions have affected the AI model’s coding performance for neurology and account for the discrepancies seen in both the Wilcoxon signed rank test and the κ coefficient test.

**Table 4.** Analysis of major diagnostic category for 366 neurology patients admitted from February to April 2023.

No.	MDC^a^	AI^b^-assisted case coding (n=366), n (%)	Cases coded by human coders (n=366), n (%)	Kappa value	*P* value
1	Diseases and disorders of the nervous system	281 (76.8)	289 (79)	0.62	<.001
2	Diseases and disorders of the eye	17 (4.6)	18 (4.9)	0.84	<.001
3	Diseases and disorders of the ear, nose, mouth, and throat	21 (5.7)	20 (5.5)	0.77	<.001
4	Diseases and disorders of the respiratory system	1 (0.3)	1 (0.3)	–0.03	.96
5	Diseases and disorders of the circulatory system	20 (5.5)	12 (3.3)	0.54	<.001
6	Diseases and disorders of the digestive system	1 (0.3)	1 (0.3)	1.00	<.001
8	Diseases and disorders of the musculoskeletal system, and connective tissue	6 (1.6)	8 (2.2)	0.56	<.001
9	Diseases and disorders of the skin, subcutaneous tissue, and breast	2 (0.6)	2 (0.6)	1.00	<.001
10	Diseases and disorders of the endocrine, nutritional, and metabolic systems	1 (0.3)	2 (0.6)	0.67	<.001
11	Diseases and disorders of the kidney and urinary tract	4 (1.1)	1 (0.3)	0.40	<.001
14	Pregnancy, childbirth, and puerperium	0 (0)	1 (0.3)	—^c^	—
16	Diseases and disorders of the blood and blood forming organs and immunological disorders	1 (0.3)	1 (0.3)	1.00	<.001
17	Myeloproliferative diseases and disorders (poorly differentiated neoplasms)	0 (0)	1 (0.3)	—	—
18	Infectious and parasitic diseases and disorders	4 (1.1)	0 (0)	—	—
19	Mental diseases and disorders	3 (0.8)	0 (0)	—	—
23	Factors influencing health status and other contacts with health services	1 (0.3)	9 (2.5)	–0.05	.87
—	None	3 (0.8)	0 (0)	—	—

a MDC: major diagnostic category.

b AI: artificial intelligence.

c Analysis was not performed when there were 0 cases in a coding group.

In the circulatory system, the statistical results for the Division of Cardiology and Division of Cardiovascular Surgery in the Wilcoxon signed rank test were also markedly different. Upon closer examination of the data from the exploratory study, it was discovered that in the Division of Cardiovascular Surgery, half of the cases helped by AI-coding modules did not have the main diagnosis coded, which could be attributed to differences in how physicians document medical records. For example, after carefully reviewing the 24 cases of data collected by the Cardiac Surgery Department, it was found that 12 Taiwan DRGs were inconsistent. All of these did not follow the disease classification coding rules and did not include the main diagnosis (Table 5).

**Table 5.** Discussion on writing medical records in the Division of Cardiovascular Surgery.

Number	Excerpt of discharge diagnosis	Cause analysis
Case 1	“Chest tightness for a week Acute heart failure with reduced ejection fraction * 2023/04/24 Thallium 201 (Stress SPECT (single-photon emission computed tomography) imaging): mild myocardial ischemia in inferolateral wall of LV (left ventricle)”	The examination results were attached to the discharge diagnosis, leading to coding confusion.
Case 2	“Type A aortic dissection - post TEVAR (Thoracic endovascular aneurysm repair)+ stenting grafts in the ascending to descending aorta, left common carotid and subcalvian arteries on 2023/02/07 # Suspect gastroparesis related to relative gastric malperfusion”	The discharge diagnosis showed coding confusion with previously treated conditions.
Case 3	“Type B dissection, intramural hemorrhage - 2023/03/10 Chest CT (computed tomography) angiography:1) Suspect intramural hematoma in the descending aorta.2) Suspect thrombus formation in the bilateral femoral arteries.3) Suspect a thrombosed aneurysm in the right internal iliac artery”	The anatomical location was not clearly documented, making correct coding impossible.

Furthermore, in MDC 14 (pregnancy, childbirth, and puerperium) and MDC 21 (injuries, poisonings, and toxic effects of drugs), there are specific coding rules. Clinical coders need to synthesize the entire medical record information and apply the coding rules, which could result in diagnoses different from those presented in the discharge summary.

### Limitations

The AI-coding module was trained on inpatient data from April 2019 to December 2020. Advancements in medical care might lead to variations in the diseases of admitted patients. Taken together, these show situations where the AI-coding assistance module might not capture the main diagnosis, as observed in the Dermatology Department.

### Conclusions

With the rapid advancements in global medical technology and the evolving challenges of diseases, the development of DRG-based hospital payment systems in various countries is also meeting significant challenges. Key areas for future research include determining the flexibility of DRG payments, balancing payment structures, and aligning with disease management goals [23]. The Taiwan DRGs system, like those in other countries, aims to prevent medical institutions from delivering excessive services and causing unnecessary waste, all while safeguarding patient rights. It looks to strengthen management mechanisms to improve the quality and efficiency of care and ensure fair payments among peers.

In this context, AI-assisted coding emerges as a powerful tool [24]. A recent study used cross-random control methods to prove that AI-assisted coding reduces the coding workload [25]. The focuses of our research were the practical applications of AI models, with two main goals. The first was to investigate the consistency between the AI-assisted coding module and coding professionals and the second was to find the departments suitable for using the AI-assisted coding module. The research results showed that the highest consistency in MDC classification was seen in diseases of the respiratory system, as well as infectious and parasitic diseases. In the analysis of various inpatient specialties, departments such as the Division of Cardiology, Division of Nephrology, and Department of Urology showed no significant difference from coder-assigned coding results; accordingly, consideration could be given to integrating the AI-assisted coding module into the hospital information system, allowing physicians to reference Taiwan DRGs assignments for hospitalized patients, thus effectively controlling medical expenses.

However, upon analyzing the entire hospital department, discrepancies were observed in alignment with disease categorizations and personnel coding, so the research team is actively working on continuous improvements. Nevertheless, AI-assisted coding indeed served as a valuable reference by reducing human errors, as during the research period, it was found that the error rate detected by human coders (number of coding errors by human coders/total cases) was 1.9% (50/2632). Given the regular updates to the tool book by the Department of Health and the revisions in coding rules, the coding assistance module undoubtedly proves to be a powerful tool.

The development of AI-assisted coding for the *ICD-10-CM* PCS is just the beginning for intelligent health care in disease classification. Many operational aspects of hospitals are closely related to the *ICD-10-CM* PCS, including inpatient coding monitoring, discharge preparation services, and infectious disease surveillance, among others. For hospital administrators, the goal of AI-assisted coding is to achieve best operational revenue. For human coders with the assistance of an *ICD-10-CM* AI coding system, work time is reduced. Additionally, strengthening knowledge in clinical documentation improvement enables human coders to maximize their role, positioning them to become documentation experts [15] and preparing them for further career development.

## Supplementary material

10.2196/59961 Multimedia Appendix 1 Distribution of *ICD-10* (*International Statistical Classification of Diseases, Tenth Revision*) codes.

## Abbreviations

AI: artificial intelligence
BERT: bidirectional encoder representations from transformers
DRG: diagnosis-related group
HAN: hierarchical attention network
*ICD*: *International Classification of Diseases*
*ICD-10*: *International Statistical Classification of Diseases, Tenth Revision*
*ICD-10-CM*: *International Classification of Diseases, Tenth Revision, Clinical Modification*
*ICD-9-CM*: *International Classification of Diseases, Ninth Revision, Clinical Modification*
MDC: major diagnostic category
NHIA: National Health Insurance Administration
PCS: procedure coding system
WHO: World Health Organization
