# Supplementary figures and images for: Application of Clinical Department–Specific AI-Assisted Coding Using Taiwan Diagnosis-Related Groups: Retrospective Validation Study
Source: JMIR Hum Factors. 2025 Feb 12;12:e59961. doi: 10.2196/59961 (PMC11838144; doi:10.2196/59961)

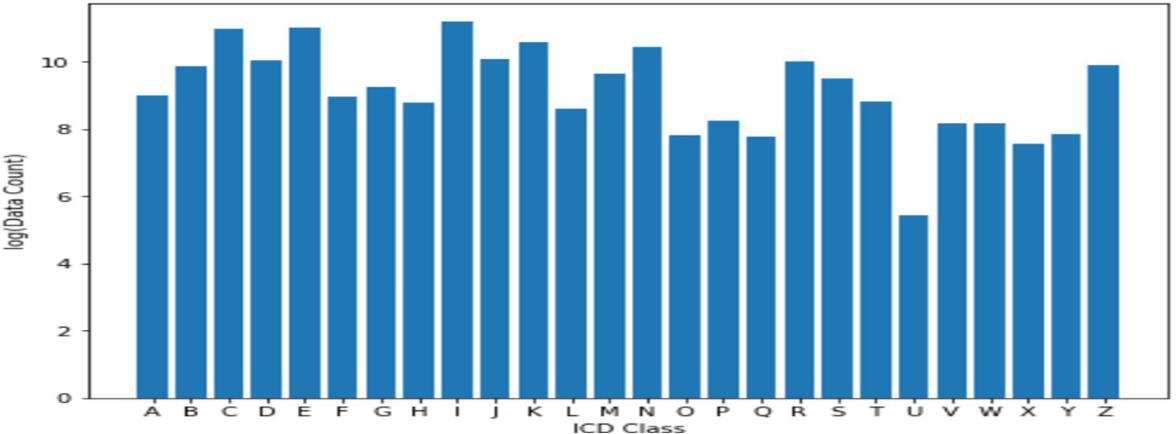

Supplement: Multimedia Appendix 1 [file humanfactors-v12-e59961-s001.jpeg]
